# Biological and Clinical Significance of Adaptive Evolution of Coronaviruses

**DOI:** 10.3390/life11111129

**Published:** 2021-10-23

**Authors:** Apostolos Beloukas, Theodoros Rampias

**Affiliations:** 1 Department of Biomedical Sciences, University of West Attica, 12243 Athens, Greece; 2 Institute of Infection and Global Health, University of Liverpool, Liverpool L69 7BE, UK; 3 Biomedical Research Foundation of the Academy of Athens, Basic Research Center, 11527 Athens, Greece

RNA viral genomes are generally small genomes that rarely exceed 10 kb in size. Moreover, these genomes are characterized by high mutation rates (typically, 10^−4^/base/replication cycle) since the viral polymerases (i.e., RNA-dependent RNA polymerase and reverse transcriptase) are prone to misincorporation and lack proofreading activity. The high mutation rate leads to high genetic variability and fast evolution, providing an adaptive potential regarding new cellular receptors and hosts [1,2]. On the other hand, reduced genome in RNA viruses restricts the accumulation of excessive numbers of deleterious mutations that would lead to a dramatic loss in fitness [3]. This pressure for genome compression is reflected by the extensive presence of overlapping genes in genomes of RNA viruses [4]. Notably, genome expansion in RNA viruses has been identified in Coronaviruses (CoVs, order Nidovirales, family Coronaviridae) that harbor the largest RNA genome identified yet identified (26–32 kb). In this Special Issue of *Life*, several researchers have studied or reviewed this group of RNA viruses with a specific focus on their ecology, evolution, and epidemiology.

A major evolutionary event in these single-stranded positive-sense (+) RNA viruses was the acquisition of ExoN exoribonuclease (Nsp14). The RNA 3′ end mismatch excision activity of this enzyme promotes replication fidelity, and genome expansion in CoVs is believed to be at least partially mediated by Nsp14 gene acquisition [5]. Mutations generated by the RNA-dependent RNA polymerase, together with recombination, horizontal gene transfer and gene duplication events, drive CoVs’ genetic diversity [6,7]. However, their larger genome, is considered to allow for extra plasticity in gaining and modifying genes from homologous recombination and gene transfer events without significant alterations in fitness. As a result, CoVs are able to explore new hosts through the interaction of different cellular receptors by their spike proteins, which are hot spots in viral CoVs’ genomes for natural selection and evolution [8].

In this context, phylogenetic analysis provides evidence that the evolution of CoVs includes several events of interspecies transmission linked to recombination events. For instance, all three novel zoonotic human CoVs (SARS-CoV, MERS, and SARS-CoV-2) have been exposed to recombination events in their evolutionary history [9,10,11]. The potential of animal coronaviruses for cross-species transmission is comprehensively discussed in this issue by Bonilauri and Rugna [12], while an update on the SADS-CoV (Swine Acute Diarrhea Syndrome Coronavirus) phylodynamics by Scarpa and colleagues is also included in our Special Issue [13].

Prior host jumps of coronaviruses to humans, bats and birds have been identified as natural animal reservoirs for coronaviruses, while civets and other mammals such as camels have been identified as intermediate amplifying hosts. For example, the emergence of Middle-East Respiratory Syndrome Coronavirus (MERS-CoV) into humans involved viral exchange between bats and camels before transmission to humans was possible [14,15,16]. In this issue, Vasilarou and colleagues, in a population genomics study, identified the plausible recombination events between the Betacoronavirus genomes in nonhuman hosts that have contributed to the evolution of SARS-CoV-2. Moreover, they investigated the positive selection processes in genomic regions that had been subjected to recombination, and provided evidence that the pangolin coronavirus genome may have contributed to the SARS-CoV-2 genome by recombination with the bat coronavirus genome [8].

Selection occurring on emerging variants of coronaviruses strongly contributes to the adaptation that occurs following a shift from one host to another. The high levels of genetic variation combined with the large population sizes of coronaviruses provide an ideal genetic landscape for selection to optimize the host colonization. Selection on mutations affecting the spike protein seems to play the most important role in adaptation process since spike protein is involved in the host cellular entry and the evasion of host cellular immune responses. However, there is evidence that selection on mutations affecting other encoded proteins also contributes to innate immune escape. In this issue, Mandilara and colleagues highlighted the important role of Nsp15 and Nsp16 RNA in modifying enzymes during the innate immune escape of coronaviruses [17].

As with other viruses, CoVs also depend on cellular host factors and pathways for successful replication. Valuable information from interactome studies led to the rapid discovery of host RNA binding proteins that bind to viral RNA, regulating its replication and translation [18]. Since SARS-CoV-2 depends on the host proteins to translate its capped and poly-adenylated RNAs, many proteins that are involved in the recognition of the poly(A) tail, such as PABPC1, PABPC4, and PABPCN1, and subunits of eukaryotic initiation complexes that are involved in cap binding (eIF4F subunits) or ribosome binding (eIF3), were retrieved in these studies. Similarly, the landscape of the SARS-CoV-2 protein–protein interactome has been resolved by several recent studies and has provided important information about the host signaling pathways hijacked by the virus in order to facilitate its life cycle. According to these studies, a significant cluster of SARS-CoV-2-interacting proteins is associated with endomembrane compartments or vesicle trafficking pathways. These host proteins facilitate virus entry, since upon attachment to human cells, SARS-CoV-2 internalize and fuse in the endosome. Intracellular membranes also have an essential role in SARS-CoV-2 genome replication as they can form anchor sites for the assembly of viral RNA replication complexes (VRCs). Moreover, during egress, SARS-CoV-2 virions follow a non- canonical lysosomal exocytosis pathway to exit cells [19]. Another group that includes innate immune signaling proteins was found to interact via SARS-CoV-2 viral proteins. This group consists of interferon and NF-κB pathway components such as the TBK1, TBKBP1, RNF41, TLE1, and NLRX1 proteins [20].

Despite the genetic diversity among different coronavirus clades and the infection of different organisms and cell types, specific host signaling pathways that drastically affect their propagation are manipulated in a common way. For instance, as reviewed by Gioti and colleagues in this issue, animal coronaviruses employ similar molecular mechanisms in order to hijack the originally protective apoptotic signaling of the host and stimulate apoptosis at later stages of infection in order to favor viral dissemination by cell breakdown [21].

During the COVID-19 pandemic, mutations of SARS-CoV-2 that are associated with high transmissibility have been subjected to positive selection (Figure 1). Several experimental studies have focused on the spike protein in order to characterize such mutations in the context of the interaction with the ACE-2 receptor or in the context of immune escape. However, less is known about the biological effect of mutations in genomic regions that encode Nsp proteins, on their interactions with the host components. In this issue, three manuscripts focus on the molecular epidemiology of SARS-CoV-2. The transmissibility of viral strains circulating in Romania during the first months of the pandemic was investigated by Surleac et al. [22], while Kostaki et al., investigated the temporal dominance of the B.1.1.7 over the B.1.354 SARS-CoV-2 variant [23], and Bousali et al. generated and analyzed the SARS-CoV-2 spatiotemporal profile of molecular transmission clusters in ten European regions during the first pandemic wave [24].

Not surprisingly, given the unfolding pandemic caused by SARS-CoV-2, this Special Issue also attracted more clinically oriented research. The significant morbidity and mortality of COVID-19, as well as the huge burden it posed to clinical services globally, led to an unprecedented global research mobilization that resulted, within just a few months, in the production of research outputs and knowledge that normally requires years to be accumulated. This intense research production came with some limitations. For example, numerous clinical trials evaluated the same treatments, unwarrantedly exposing numerous patients to experimental treatments that were later proved to be ineffective or associated with adverse events (e.g., hydroxychloroquine or lopinavir/ritonavir). Moreover, other clinical trials omitted endpoints that are important to patients [25]. In this issue, Mathioudakis et al. present a systematic review evaluating the endpoints assessed in ongoing and completed COVID-19 trials, as well as the instruments used to measure them [26]. The results of this study could inform consensus on the selection of the most important endpoints for assessment in future trials, ensuring their results are clinically relevant and comparable. On the other hand, Ulinici and colleagues summarized, in a comprehensive review, the current data on screening, diagnostic, and prognostic tests for COVID-19, not only providing a great reference for clinicians, but also revealing research gaps that should be remedied in future studies [27]. The evaluation of the seroprevalence of antibodies against SARS-CoV-2 in a large cohort of personnel and students at the University of Athens, conducted by Tsitsilonis and colleagues, revealed insights around the prevalence of asymptomatic infection across different ages, providing data that could be used to guide health policy [28]. Last but not least, Mathioudakis and colleagues, presented the first real-life data around the safety and reactogenicity of the mRNA versus viral vector COVID-19 vaccines, from a well-conducted electronic survey with over 2000 respondents [29]. This study confirms the safety of both vaccine types and reveals a higher prevalence of side effects among younger patients and those with previous exposure to COVID-19 (through the infection or vaccine).

In summary, this Special Issue represents a collection of articles that study and discuss different aspects of the ecology, evolution, and epidemiology of coronaviruses, as well as evolving research on clinical aspects on COVID-19. We hope that the diverse articles of this issue cover a significant part of coronavirus research in these fields. A better understanding of the molecular mechanisms that govern genetic evolution and adaptation will help us to develop a better epidemiological surveillance and battle the coronavirus-driven threats to public health more efficiently. It is obvious that more remains to be discovered about the manipulation of cellular signaling pathways by the viral proteins. The limitations of our knowledge on the immune escape mechanisms of coronaviruses pose challenges for the next years in terms of growing a more comprehensive understanding of these adaptation processes and developing new therapeutic approaches.

## Figures and Tables

**Figure 1:**
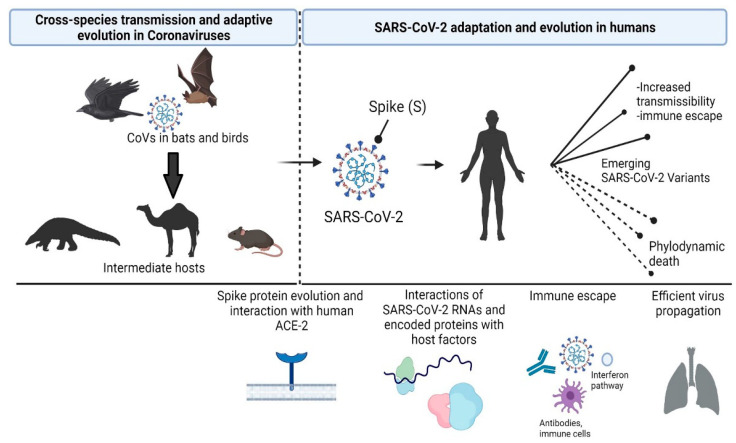
Evolution of Coronaviruses and SARS-CoV-2 adaptation in humans. The potential for cross-species transmission is a hallmark of adaptive evolution in coronaviruses. Selected mutations and/or recombination events were associated with the potential of SARS-CoV-2 to infect human cells, hijack host cell signaling pathways, and escape the immune system. The mutation landscape of emerging SARS-CoV-2 variants has different fitness effects that, in turn, affect the transmission dynamics.
